# Partial Solvation Parameters of Drugs as a New Thermodynamic Tool for Pharmaceutics

**DOI:** 10.3390/pharmaceutics11010017

**Published:** 2019-01-04

**Authors:** Andreas Niederquell, Nicole Wyttenbach, Martin Kuentz, Costas Panayiotou

**Affiliations:** 1 Institute of Pharma Technology, University of Applied Sciences and Arts Northwestern Switzerland, Hofackerstr. 30, 4132 Muttenz, Switzerland; andreas.niederquell@fhnw.ch; 2 Roche Pharmaceutical Research & Early Development, Pre-Clinical CMC, Roche Innovation Center Basel, F. Hoffmann-La Roche Ltd., Grenzacherstr. 124, 4058 Basel, Switzerland; nicole.wyttenbach@roche.com; 3 Department of Chemical Engineering, University of Thessaloniki, 54124 Thessaloniki, Greece; cpanayio@cheng.auth.gr

**Keywords:** drug, partial solvation parameters, solubility, surface energy, thermodynamics

## Abstract

Partial solvation parameters (PSP) have much in common with the Hansen solubility parameter or with a linear solvation energy relationship (LSER), but there are advantages based on the sound thermodynamic basis. It is, therefore, surprising that PSP has so far not been harnessed in pharmaceutics for the selection of excipients or property estimation of formulations and their components. This work introduces PSP calculation for drugs, where the raw data were obtained from inverse gas chromatography. It was shown that only a few probe gases were needed to get reasonable estimates of the drug PSPs. Interestingly, an alternative calculation of LSER parameters in silico did not reflect the experimentally obtained activity coefficients for all probe gases as well, which was attributed to the complexity of the drug structures. The experimental PSPs were proven to be helpful in predicting drug solubility in various solvents and the PSP framework allowed calculation of the different surface energy contributions. A specific benefit of PSP is that parameters can be readily converted to either classical solubility or LSER parameters. Therefore, PSP is not just about a new definition of solvatochromic parameters, but the underlying thermodynamics provides a unified approach, which holds much promise for broad applications in pharmaceutics.

## 1. Introduction

The majority of drug formulations are complex multicomponent and often multiphase systems, and empirical approaches have traditionally been applied in the field of pharmaceutics. While more rigorous thermodynamic treatments were mostly limited to rather simple model formulations, it is especially the solubility parameter concept and in particular the Hansen solubility parameter (HSP) [1,2] that has been widely applied to select pharmaceutical excipients. These applications in pharmaceutics focusing on poorly water-soluble drugs have been reviewed recently [3]. The rather minimal theoretical nature of the Hildebrand solubility parameter and of the more versatile HSP comes with the disadvantage of limitations owing to the given simplifications [4]. An example here is hydrogen bonding, where, although the interaction is considered by the HSP (unlike the Hildebrand solubility parameter), there is no differentiation between the acidity and basicity of a molecule. However, such a proton donation/acceptance tendency is taken into account by linear free energy relationships (LFER) that were greatly promoted through the research of Professor Abraham [5,6]. Molecular predictors can be obtained from a database [7] and diverse pharmaceutical applications of LFER have been reported over the last two decades. LFER applications with a primary focus on a solvation step are also named as a linear solvation energy relationship (LSER). Notable are examples of partitioning between water and typical pharmaceutical solvents [8,9,10] or the prediction of aqueous solubility [11]. Other applications are about partitioning from water into colloidal (pseudo)phases, like liposomes or mixed micelles of a simulated intestinal medium [12,13]. Abraham solvation predictors have been further employed in quantitative structure property relationships (QSPR), such as human intestinal absorption or drug partitioning from blood into the brain [14,15].

Very similar to HSP and LFER is the so-called partial solvation parameter (PSP) approach [16,17,18,19,20,21]. Interestingly, PSP has, to our best knowledge, so far not been applied in pharmaceutics, even though this approach has distinct advantages. PSP shares the versatility of HSP and LSER but has key advantages similar to the conductor-like screening model for real solvents (COSMO-RS) [22,23,24,25,26,27,28,29] and equation-of-state [30,31,32,33,34] approaches. This similarity is important, since COSMO-RS [22,23,24,25,26,27,28,29] has been applied already for cocrystal screening and the solubility of various drugs and drug-like compounds (e.g., amides, methylxanthines, or phenolic acids), for drug solubility in ionic liquids (e.g., methotrexate or flavonoids), for excipient selection, and various other applications. Since PSP is developed with a sound thermodynamic basis, it is well suited for the integral and coherent characterization of materials and the prediction of their behavior in bulk phases and interfaces.

Initially [16,17,18], PSPs were heavily based on the quantum-mechanics-based COSMO-RS model [22,23,24,25,26,27,28,29], one of the most successful predictive thermodynamic models currently in use. More specifically, the moments (or COSMOments) of the distribution profiles of molecular screening surface charges (or *σ*-profiles) of COSMO-RS [23] were used as molecular descriptors for the estimation of PSPs. This would require access to the COSMObase [24] or to quantum chemical calculation suites, such as TURBOMOLE or DMol3 [25,26]. Recently [7], Abraham’s LSER descriptors became freely available for a large number of compounds. PSPs were, thus, redefined in terms of LSER descriptors in order to take advantage of this large and freely accessible inventory [20,21].

PSPs are not just an alternative set of molecular descriptors. They are, primarily, a novel concept aiming at interconnecting diverse QSPR-type approaches and databases of molecular descriptors and intermolecular interactions to bring this valuable information on a common denominator, thereby facilitating the transfer and conversion of molecular information. In contrast to HSP and LSER approaches, PSP is a coherent thermodynamic model for pure fluids and mixtures and for bulk phases and interfaces. PSPs have been used already for the prediction of vapor–liquid and solid–liquid phase equilibria, the characterization of high polymers, and the prediction of polymer–polymer miscibility and the wetting behavior of polymeric solid surfaces [16,17,18,19,20,21].

Despite the multiple opportunities offered by PSP, this approach has so far not found its way to pharmaceutics. Therefore, the present study aimed at LSER and PSP determination of selected drugs based on inverse gas chromatography data. The new LSER descriptors are, in addition, corrected for self-association—a feature disregarded in the original LSER approach and database [7]. An experimental comparison was made for drug solubility in a series of solvents and, furthermore, the components of drug surface energy were considered.

## 2. Theory of the Partial Solvation Parameter (PSP) Approach

In this subsection, we summarize the definitions, the working equations, and the interrelations between partial solvation parameters (PSP) and LSER molecular descriptors. Details may be found in the recent references [17,20,21], along with extensive tables with all the needed LSER descriptors.

### 2.1. Definitions

The dispersion PSP, *σ_d_*, reflects hydrophobicity, cavity effects, and dispersion or weak nonpolar interactions. It maps the McGowan volume, *V*_x_, and excess refractivity, *E*, LSER descriptors [5,6,7] of the compound as follows [20,21]:

(1) $$\sigma_{d}=100\sqrt{\frac{3.1V_{x}+E}{V_{m}}};\;(\mathrm{dispersion}\;\mathrm{PSP}),$$

where *V_m_* is the molar volume of the compound.

The polarity PSP, *σ_p_*, reflects dipolar (Debye-type as well as Keesom-type) interactions. It maps the polarity, *S*, LSER descriptor [5,6,7] of the compound according to the following equation [20,21]:

(2) $$\sigma_{p}=100\sqrt{\frac{S}{V_{m}}};\;(\mathrm{polarity}\;\mathrm{PSP}).$$

The acidity and basicity LSER descriptors, *A* and *B* are mapped into the corresponding PSPs as follows [20,21]:

(3) $$\sigma_{Ga}=100\sqrt{\frac{A}{V_{m}}};\;(\mathrm{acidity}\;\mathrm{PSP}),$$

(4) $$\sigma_{Gb}=100\sqrt{\frac{B}{V_{m}}};\;(\mathrm{basicity}\;\mathrm{PSP}).$$

These descriptors reflect the stronger or specific interactions of the hydrogen-bonding type or the Lewis acid/base interactions. It is important to stress the point that these two PSPs are Gibbs free-energy descriptors (indicated by the subscript, *G*). As such, they give the Gibbs free energy change directly upon formation of the hydrogen bond (or the acid–base interaction):

(5) $$-G_{HB,298}=2V_{m}\sigma_{Ga}\sigma_{Gb}=20000\sqrt{AB}.$$

This quantity is related to the energy (enthalpy, essentially, for low to moderate pressures) change by the classical equation:

*G_HB_* = *E_HB_* − *TS_HB_*, (6)

where *S_HB_* is the corresponding entropy change. Generally, *E_HB_* and *S_HB_* vary independently. However, a good approximation may be obtained by adopting the value of *S_HB_* = −26.5 JK^−1^mol^−1^, known to be valid for lower alkanols (of corresponding average *E_HB_* = −23,000 Jmol^−1^) as a reference value [19,20,21]. Certainly, *E_HB_* and *G_HB_* are equal to zero when *A* and/or *B* are zero. Assuming linear variations and combining with Equation (5), we obtain the following working equations for the hydrogen bonding quantities:

(7) $$E_{HB}=-30,450\sqrt{AB},$$

and:

(8) $$S_{HB}=-35.1\sqrt{AB}.$$

Then, an equation for the free energy change at any temperature can be formulated:

(9) $$G_{HB}=-\left(30,450-35.1T\right)\sqrt{AB}.$$

When the molecule has one hydrogen-bonding donor and one hydrogen-bonding acceptor, the total number of hydrogen bonds per mol in the system is given by:

(10) $$\frac{N_{11}}{N}=\frac{A_{11}+2-\sqrt{A_{11}\left(A_{11}+4\right)}}{2}=\frac{A_{11}+2-\sqrt{{\left(A_{11}+2\right)}^{2}-4}}{2}=r_{1}\nu_{11},$$

where:

(11) $$A_{11}=r_{1}\exp\left(G_{HB}^{}/RT\right)=r_{1}/K_{11},$$

and *r*_1_ is the number of segments for molecule 1 obtained, typically, via the universal quasichemical UNIQUAC/UNIFAC group-contribution method [35,36]. In this work, the much simpler equation [21]:

(12) $$r_{1}=\frac{V_{X}}{0.213},$$

is adopted. If s_1_ is the “surface-to-volume” ratio of the molecule [35,36], the external intermolecular contacts per molecule are 10*q*_1_ = 10*r*_1_*s*_1_.

With the above definitions, one may obtain the hydrogen-bonding contribution to the cohesive energy density as follows [34]:

(13) $$ced_{HB}=-\frac{r_{1}\nu_{11}E_{HB}}{V_{m}}.$$

### 2.2. Mixture Thermodynamics

In a binary mixture of *N*_1_ and *N*_2_ moles of components 1 and 2, respectively, the mole fraction, *x*_1_, the volume fraction, *φ*_1_, and the site or surface area fraction, *θ*_1_, are defined classically as follows:

(14) $$x_{1}=\frac{N_{1}}{N_{1}+N_{2}}=\frac{N_{1}}{N};\;\;\varphi_{1}=\frac{x_{1}r_{1}}{x_{1}r_{1}+x_{2}r_{2}}=\frac{x_{1}r_{1}}{r_{}};\;\;\theta_{1}=\frac{x_{1}q_{1}}{x_{1}q_{1}+x_{2}q_{2}}=\frac{x_{1}q_{1}}{q_{}},$$

and, similarly, for component 2. 

The activity coefficient is considered here to be a product of the combinatorial and residual contributions. For the combinatorial contribution to the activity coefficient (superscript, C), one may use either the classical Guggenheim–Staverman expression [37,38] or the simpler widely used Flory–Huggins equation [39]:

(15) $$\ln\gamma_{1}^{c}=\ln\frac{\varphi_{1}}{x_{1}}+\left(1-\frac{r_{1}}{r_{2}}\right)\varphi_{2}.$$

In the LSER/PSP framework, we have three separate contributions to the residual activity coefficient arising from the dispersion, the polar, and the hydrogen-bonding or acid/base intermolecular interactions. The first two contributions are of the classical quadratic form of solubility parameter or cohesive energy density differences [1,2,8,40] and take the following form [17,18,19,20,21]:

(16) $$\ln\gamma_{1}^{VE}=\frac{10000V_{x,1}\varphi_{2}^{2}}{RT}{\left(\sqrt{3.1+\frac{E_{1}}{V_{x,1}}}-\sqrt{3.1+\frac{E_{2}}{V_{x,2}}}\right)}^{2},$$

and:

(17) $$\ln\gamma_{1}^{S}=\frac{10000V_{x,1}\varphi_{2}^{2}}{RT}{\left(\sqrt{\frac{S_{1}}{V_{x,1}}}-\sqrt{\frac{S_{2}}{V_{x,2}}}\right)}^{2}.$$

As observed, these two contributions are always positive. Negative contributions (*γ* values less than unity) may arise from the third contribution only, which arises from the hydrogen-bonding (or Lewis acid/base) interactions in the system. In the general case of solvent with one donor and one acceptor group and a solute with also one proton and one acceptor group, the hydrogen-bonding contribution to the activity coefficient is obtained through Veytsman’s statistics [41,42,43] and reads:

(18) $$\ln\gamma_{1}^{H}={\left[r_{1}\nu_{H}-\ln\frac{x_{1}}{x_{1}-r_{1}\nu_{11}-r_{1}\nu_{12}}-\ln\frac{x_{1}}{x_{1}-r_{1}\nu_{11}-r_{1}\nu_{21}}\right]}_{mixture}-{\left[r_{1}\nu_{11}+2\ln\left(1-r_{1}\nu_{11}\right)\right]}_{pure1}.$$

The *ν_ij_* in Equation (18) are the reduced numbers of hydrogen bonds in the system between donors of type *i* and acceptors of type *j*, or (cf. Equation (10)):

(19) $$\nu_{ij}=\frac{N_{ij}^{}}{rN},$$

and:

(20) $$\nu_{H}=\nu_{11}+\nu_{12}+\nu_{21}+\nu_{22}.$$

These reduced quantities may be obtained by solving, with a simple iterative scheme, the following free-energy minimization conditions [17,41,42,43]:

(21) $$\frac{\nu_{ij}}{\nu_{i0}\nu_{0j}}=\exp\left(-\frac{G_{HB,ij}^{}}{RT}\right)(\mathrm{for}\;\mathrm{all}\;i,\;j\;\mathrm{pairs}).$$

*ν_i_*_0_ and *ν*_0*j*_ are the reduced numbers of nonhydrogen bonded donors *i* and acceptors *j*, respectively, or:

(22) $$\begin{array}{l} r\nu_{i0}=x_{i}-r\nu_{ii}-r\nu_{ij}, \end{array}$$

(23) $$\begin{array}{l} r\nu_{0j}=x_{j}-\nu_{ij}-\nu_{jj}. \end{array}$$

*G_HB,ij_* in Equation (21) is the free-energy change upon formation of an *i*–*j* hydrogen bond. It is this quantity which requires the knowledge of the hydrogen-bonding LSER descriptors, *A_i_* and *B_j_* (cf. Equation (9)).

Combining Equations (16)–(18), we obtain the following working equation for the residual activity coefficient or the Flory–Huggins *χ*_12_ interaction parameter:

(24) $$\begin{array}{l} \ln\gamma_{1}^{res}=\ln\left(\gamma_{1}^{VE}\gamma_{1}^{S}\gamma_{1}^{H}\right)=\\ =\frac{10000V_{x,1}\phi_{2}^{2}}{RT}\left\{{\left(\sqrt{3.1+\frac{E_{1}}{V_{x,1}}}-\sqrt{3.1+\frac{E_{2}}{V_{x,2}}}\right)}^{2}+{\left(\sqrt{\frac{S_{1}}{V_{x,1}}}-\sqrt{\frac{S_{2}}{V_{x,2}}}\right)}^{2}\right\}+\\ +{\left[r_{1}\nu_{H}-\ln\frac{x_{1}}{\nu_{10}}-\ln\frac{x_{1}}{\nu_{01}}\right]}_{mixt}-{\left[r_{1}\nu_{11}+2\ln\left(1-r_{1}\nu_{11}\right)\right]}_{pure1}=r_{1}\chi_{12}\phi_{2}^{2} \end{array}$$

This is a most useful equation in handling phase equilibria and related properties of mixtures. It may also be used for the prediction of drug solubility. The solubility (mole fraction) of solid 1 (drug) in solvent 2 may be obtained from the classical (though approximate) solid–liquid equilibrium Equation (25) [44,45,46]:

(25) $$y_{1}=\frac{1}{\gamma_{1}^{c}\gamma_{1}^{res}}\exp\left\{\frac{\Delta H_{1}^{m}}{RT}\left(\frac{T}{T_{1}^{m}}-1\right)\right\},$$

where Δ*H*_1_*^m^* is the enthalpy of fusion of solid 1 and *T*_1_*^m^* its melting point.

### 2.3. Surface Energy Components and Wetting Phenomena

For pure compounds, the defining equation for the hydrogen-bonding surface tension, *γ_hb_*, is [18,20,21,47,48]:

(26) $$\gamma_{hb}=2\sqrt{\gamma_{a}\gamma_{b}}.$$

The acidic and basic surface tension components, *γ_a_* and *γ_b_*, respectively satisfy the following equation [20,21]:

(27) $$\frac{\gamma_{a}}{A}=\frac{\gamma_{b}}{B}=\frac{\gamma_{VES}}{3.1V_{x}+E+S}.$$

The nonhydrogen-bonding component, *γ_VES_*, of the surface tension, when added to the hydrogen-bonding component, give the total surface tension, *γ*, of the compound, or:

(28) $$\gamma =\gamma_{VES}+\gamma_{hb}=\gamma_{V}+\gamma_{E}+\gamma_{S}+2\sqrt{\gamma_{a}\gamma_{b}}.$$

Equations (26)–(28), when combined, lead to the following working equation for *γ_VES_*:

(29) $$\gamma_{VES}=\gamma \frac{3.1V_{x}+E+S}{3.1V_{x}+E+S+2\sqrt{AB}}.$$

The acidic and basic surface tension components, *γ_a_* and *γ_b_*, may then be obtained by combining Equations (27) and (29).

A very common way to characterize solid surfaces is through the measurement of contact angles of a series of solvents of varying polarity with the surface. From the work of adhesion of a solid (subscript *S*), with a liquid–probe (subscript *L*) forming a contact angle, *θ*, with the solid surface, we obtain the following working equation for the contact angle [18,19,20,21,47,48]:

(30) $$\gamma_{L}\left(1+\cos\theta \right)=2\left\{\sqrt{\gamma_{VES,L}\gamma_{VES,S}}+\sqrt{\gamma_{a,L}\gamma_{b,S}}+\sqrt{\gamma_{a,S}\gamma_{b,L}}\right\}.$$

This is a most useful equation for the characterization of solid drugs and drug surfaces.

## 3. Materials and Experimental Methods

### 3.1. Materials

The compounds carvedilol, cyclosporine A, loratadine, simvastatin, and zafirlukast were purchased from Carbosynth Ltd. (Compton—Berkshire, UK), while ketoconazole was obtained from AK Scientific Inc. (Union City, CA, USA). Additionally, the analytical probe gases (decane, nonane, octane, acetone, acetonitrile, ethyl acetate, dichloromethane, methanol, as well as ethanol) and silanized glass wool were purchased from Sigma-Aldrich (Buchs, Switzerland). Heptane was bought from J.T. Baker (Deventer, the Netherlands).

The different drugs were used as received with the exception of analysis by inverse gas chromatography (IGC). The different compounds were here first converted to amorphous state by melting 2 g samples in a stainless-steel cup using a heating chamber from Binder Ltd. (Tuttlingen, Germany). Temperature was selected specifically for each drug 15 °C degrees below the melting point and then stepwise increased until liquefaction was observed. In the liquid state, the compounds were then immediately quenched in liquid nitrogen and a powder was obtained by manual milling using a mortar. All samples were stored at room temperature (24 ± 2 °C) in a desiccator at low relative humidity. Successful amorphization was confirmed by X-ray powder diffraction (XRPD) measurements (D2 Phaser benchtop X-ray diffractometer from Bruker AXS Corp., Karlsruhe, Germany).

### 3.2. Differential Scanning Calorimetry

Differential scanning calorimetry (DSC) thermograms were obtained using a DSC 1 instrument from Mettler-Toledo AG (Greifensee, Switzerland). The instrument was calibrated for temperature and enthalpy using indium. Nitrogen was used as protective gas (150 mL/min). Samples (2–3 mg) were placed in 40 μL aluminum pans with pierced aluminum lids (Mettler-Toledo AG, Greifensee, Switzerland). The melting onset temperature (*T_m_*) and the enthalpy of fusion (Δ*H_f_*) were determined by heating the samples at 10 °C/min from 25 °C to a maximum temperature of 300 °C. All DSC measurements were carried out in triplicate.

### 3.3. Inverse Gas Chromatography

Inverse gas chromatography (IGC) was used to analyze solid samples packed into a chromatographic column as stationary phase. The equipment was a surface energy analyzer (iGC-SEA) from Surface Measurement Systems Ltd. (London, UK) and about 200 mg of each drug was inserted into silanized glass columns (3 mm inner diameter) by employing a standardized packing method with silanized glass wool that was used at both ends of the column as fixation. Prior to each measurement, the samples were conditioned for 1 h at 30 °C with dry helium with 0% relative humidity at a flow rate of 15 standard cubic centimeters per minute (sccm). Dead volume of the system was determined by injecting the inert probe methane prior and after each measurement. This stationary phase was flushed with an inert carrier gas—helium in the present study. Measuring conditions were the same as applied for sample conditioning, except for a flow rate of 10 sccm that was used. For all experiments, a relative surface coverage of 5% was selected.

The extent of interactions between the solid phase of interest and the probe gas is obtained by the net retention volume *V_N_*:

(31) $$V_{N}=\frac{j}{m}F\left(t_{R}-t_{0}\right)\frac{T}{273.15},$$

where *T* is the column temperature, *F* is the carrier gas flow rate at 1 atm and 273.15 K, *m* is the sample mass, *t_R_* is the retention time of the absorbed probe gas and *t*_0_ is the mobile phase hold up time, and finally, *j* represents the James–Martin correction (that adjusts retention time for the pressure drop effect in the column bed). The calculated retention volume *V_N_* is then used to estimate the mass activity coefficient, *Ω* at infinite dilution:

(32) $$ln\left(\Omega \right)=ln\left(\frac{273.15R}{p_{0}M_{1}V_{N}}\right)-\frac{p_{0}\left(B_{11}-V_{1}\right)}{RT},$$

where *p_o_* is the saturation pressure and *M* is the molecular mass, while *B*_11_ is the second virial coefficient and *V*_1_ holds for molar volume of the probe gas. Conversion of the mass activity coefficient *Ω* to mole fraction activity coefficient *γ* (at infinite dilution) is done via the following equation:

(33) $$\ln\gamma_{1}^{res,\infty }=\ln\left(\Omega_{1}\frac{M_{1}}{M_{2}}\right)-\left(\ln\frac{r_{1}}{r_{2}}+1-\frac{r_{1}}{r_{2}}\right)=r_{1}\chi_{12}.$$

and provided the basis for further thermodynamic analysis, as described in the Results section.

### 3.4. Solubility Measurements and X-ray Analysis of Residual Solid

The solubility of compounds was determined in acetone, ethanol, acetonitrile, heptane, and dichloromethane. All experiments were performed in triplicate. The drug concentration in the solvent bulk phase was determined simultaneously with a residual solid analysis of the drug by means of XRPD to detect possible solvent-mediated phase changes. Such residual solid analysis has been published before for a different miniaturized assay [49]. In brief, an excess of drug powder (approximately 500 mg), a magnetic stir bar, and about 3 mL of vehicle were added into 5 mL glass vials with screw caps. The closed vials were equilibrated for 24 h by magnetic stirring at room temperature. Subsequently, 400 μL samples were withdrawn and filtered with Ultrafree-MC^®^ centrifugal filter devices (0.45 µm Durapore polyvinylidene fluoride (PVDF) membranes, Millipore, Bedford, MA, USA). Collected filtrates were immediately diluted with *N*-methyl-2-pyrrolidone and drug content was determined using a Waters Acquity ultra performance liquid chromatographic (UPLC) system equipped with a 2996 Photodiode Array Detector and an Acquity UPLC BEH C18 column (2.1 × 50 mm, 1.7 μm particle size) from Waters (Milford, CT, USA). The mobile phase consisted of 0.1% formic acid in de-ionized water (solvent A) and 0.1% formic acid in acetonitrile (solvent B). An isocratic flow of a mixture of solvent A and solvent B was initially applied for 0.3 min at a flow rate of 0.75 mL/min. Subsequently, the following gradient was applied for almost all compounds (except for cyclosporine A): The concentration of solvent B was linearly increased to 100% within 0.5 min. In the case of cyclosporine A, the concentration of solvent B was linearly increased to 100% within 4.3 min. Table 1 summarizes the experimental conditions (initial composition of mobile phase, detection wave length) used for the different drugs. For solid state analysis, approx. 10–20 mg of the residual wet solids was manually transferred into a 96-well MultiScreen^®^ Solubility Filter Plate (Millipore, Bedford, MA, USA) by a spatula. The filter plate was immediately sealed with adhesive acetate foil for microtest well plates (Sarstedt Inc., Newton, NC, USA) to minimize solvent evaporation. Solid state analysis of residual solids was based on a high-throughput XRPD method using a STOE Stadi P Combi diffractometer with a primary Ge-monochromator (Cu Kα radiation), imaging plate position sensitive detector (IP-PSD), and a 96-well sample stage as used in the previously published solubility residual solid state (SORESOS) assay [49]. The IP-PSD allowed simultaneous recording of the diffraction pattern on both sides of the primary beam, which were summed up by the software STOE WinXPOW to reduce effects related to poor crystal orientation statistics. Samples were analyzed directly in the 96-well filter plate with an exposure time of 5 min per well.

### 3.5. Software Used for LSER Calculation and Statistical Data Evaluation

Molecular modeling was conducted using the Absolv prediction module (V.2016 release) that is part of the ACD software Percepta (Advanced Chemistry Development Inc., Toronto, ON, Canada). The different drugs were taken from the software database or imported in the form of SMILES (simplified molecular-input line-entry system). The obtained LSER parameters are labelled as “ACD” in this article to differentiate from LSER values determined by the PSP approach.

Regression analysis and statistical testing of model validity were based on the software program Statgraphics Centurion XVI ed. Professional (V. 16.2.04) from Statpoint Technologies Inc. (Warrenton, VA, USA).

## 4. Results and Discussion

### 4.1. PSP of Solvents

A first step to apply the PSP model is to obtain the LSER descriptors of the compounds in given mixtures. Since LSER descriptors have been determined for many common solvents and other additives, it is often possible to use tabulated values from the literature. Some example values are listed in Table 2, which are widely used as solvents or probe gases in measurements of inverse gas chromatography (IGC).

The PSP approach was used to characterize the studied drugs, that is, to determine their LSER descriptors from the experimental IGC data. The McGowan volume, *V_x_*, is an atom-specific quantity and, thus, it is obtained either from the ACD software or from the freely accessible database [7] via their SMILES form. The *E* and *S* descriptors are first obtained from IGC data (*Ω*_1_ or *χ*_12_ parameter data) with alkane and aromatic hydrocarbon probes correlated with Equation (24), which, in the infinite dilution limit (*φ*_2_ → 1, *x*_2_ → 1 of IGC measurements, takes the following analytical form:

(34) $$\begin{array}{l} \ln\gamma_{1}^{res}=r_{1}\chi_{12}=\\ =\frac{10000V_{x,1}}{RT}\left\{{\left(\sqrt{3.1+\frac{E_{1}}{V_{x,1}}}-\sqrt{3.1+\frac{E_{2}}{V_{x,2}}}\right)}^{2}+{\left(\sqrt{\frac{S_{1}}{V_{x,1}}}-\sqrt{\frac{S_{2}}{V_{x,2}}}\right)}^{2}\right\}+\\ r_{1}\nu_{22}-\ln\frac{1+A_{12}-r_{2}\nu_{22}}{A_{12}}-\ln\frac{1+A_{21}-r_{2}\nu_{22}}{A_{21}}-\left\{r_{1}\nu_{11}+2\ln\left(1-r_{1}\nu_{11}\right)\right\} \end{array}$$

where:

(35) $$A_{ij}=r_{2}\exp\left(G_{HB,ij}^{}/RT\right).$$

*A* and *B* descriptors were then obtained from IGC data with hydrogen-bonding probes (acidic, like chloroform, basic, like acetone, and homosolvated, like ethanol) correlated with Equation (34).

### 4.2. Applications to Pharmaceutical Drugs

Six model drugs were selected that represent rather poorly water-soluble compounds due to their importance in pharmaceutical sciences.

The LSER descriptors for the drugs studied in this work may be estimated using the ACD software (ACD Percepta, Absolv. V.2016) and obtained values are reported in Table 3. These values were compared with determined LSER descriptors using the PSP approach based on the experimental IGC measurements.

**Figure 1:**
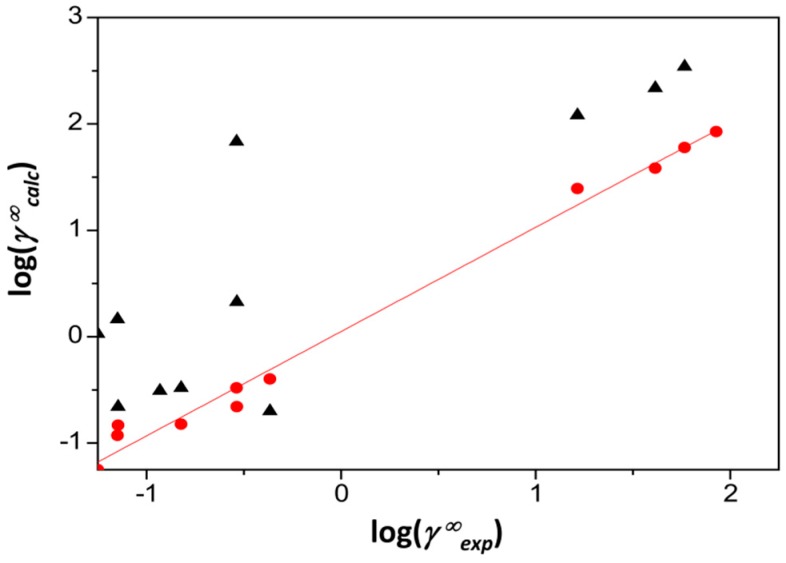
Logarithmic plot of calculated vs. experimental activity coefficients at infinite dilution (*γ∞_calc_* and *γ∞_exp_*, respectively) of various probes in cyclosporine A. The straight line is the diagonal. The circles correspond to calculations with PSP/LSER descriptors, while the triangles to calculations with ACD/LSER descriptors. Details are given in the text.

**Figure 2:**
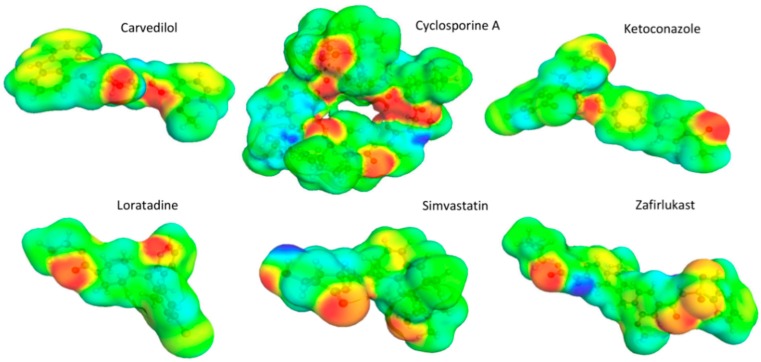
Structures of the model drugs with screening charge densities displayed. Hydrogen bonding acceptors are displayed as red, whereas the strong donor moieties of the molecules are given in blue.

In Figure 1, the experimental activity coefficients at infinite dilution are compared to calculated estimates for cyclosporine A. As observed, there is a rather significant discrepancy between experimental data and calculations when using the ACD/LSER descriptors. The picture is even worse for the other five studied drugs. Indeed, due to the rather complex structure of these drugs, it was not expected to have very precise estimations of their LSER descriptors by such a pure in silico approach as used by the ACD software. Figure 2 displays the molecular structures and screening charge densities of the different model compounds. The compounds are typically larger and more complex than most solvent molecules; they also exhibit dipolar and hydrogen-bonding capabilities. Larger molecules have, compared to rather small solvents, more options for specific effects of the three-dimensional (3D) conformation, such as shielding of moieties that are relevant for interaction. This is neglected by the 2D input structure of the ACD software. Supramolecular effects and self-association can further complicate the situation, thereby leading to poor estimates of the LSER descriptors of drugs using the 2D in silico method.

As observed in Figure 1, the PSP calculations are made much closer to the diagonal (equal experimental and calculated data) compared to the ACD values, which are significantly off the diagonal.

**Figure 3:**
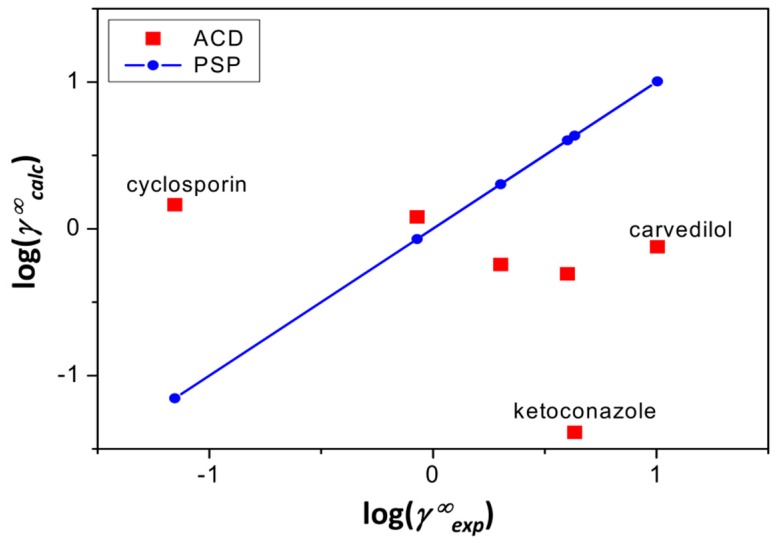
Logarithmic plot of calculated vs. experimental activity coefficients (*γ∞_calc_* and *γ∞_exp_,* respectively), of methanol at infinite dilution in the studied drugs. The straight line through the PSP calculations is the diagonal. Specific drugs were only labeled for those where the discrepancy was especially pronounced.

Relevant deviations are observed with the IGC data for all probe gases studied. Figure 3 displays the experimental and calculated activity coefficients at infinite dilution of methanol (the most hydrophilic solute/probe) for the studied drugs. In this case, the PSP calculations practically coincide with the diagonal, while the calculations with ACD/LSER descriptors are often significantly off the diagonal. However, with the exception of methanol, n-alkanes, and a couple of other solutes/probes, the IGC data could not be reconciled satisfactorily, either by the ACD or by the PSP/LSER descriptors. Thus, the reported LSER descriptors in Table 3 should be considered as initial or tentative estimates. It should be kept in mind that there can be issues of individual probe gases on an experimental level, such as association in the gas phase and deviation from the infinite dilution conditions. Analysis of further probe gases can further improve estimates of PSP/LSER descriptors determined by IGC.

**Figure 4:**
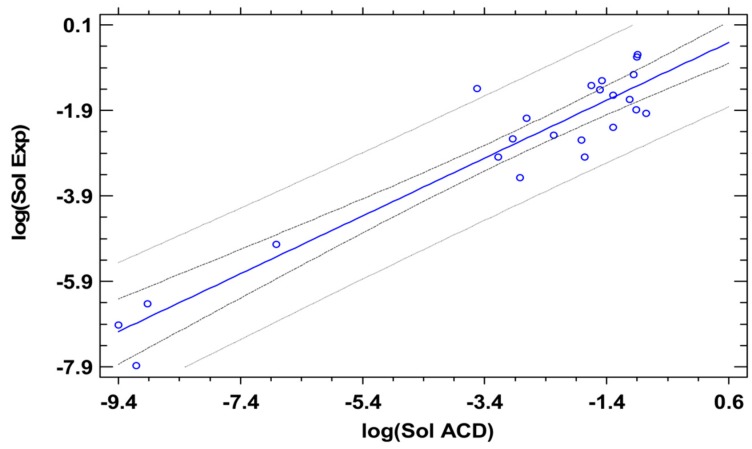
Experimental solubility, log(Sol Exp) is plotted against the solubility as estimated by the LSER approach using the ACD in silico estimation. The 95% confidence and prediction limits are shown as gray lines, while the linear model and data points are shown in blue.

**Figure 5:**
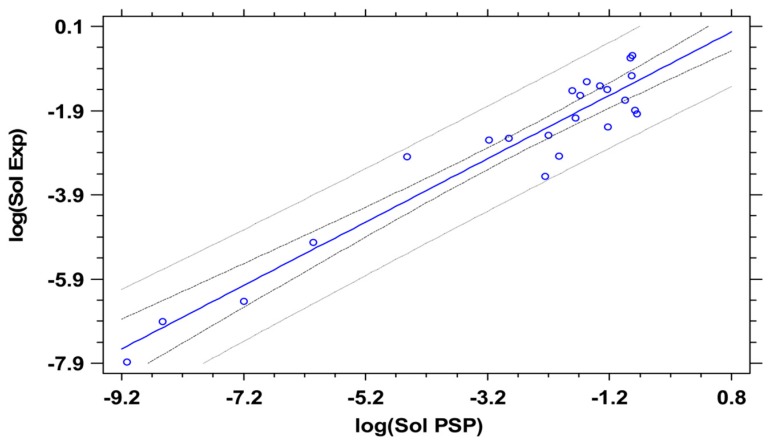
Experimental solubility, log(Sol Exp) is plotted against the calculated solubility by the LSER PSP approach. The 95% confidence and prediction limits are shown as gray lines, while the linear model and data points are in blue.

Having determined the LSER descriptors of drugs, Equation (25) can be used for the prediction of their solubilities in the various solvents. The required melting points and heats of fusion of the drugs are reported in Table 4. The predictions with both sets of the LSER descriptors (ACD and PSP) are shown in Figure 4 and Figure 5, along with the experimental solubilities. Both sets of descriptors were used with the PSP thermodynamic framework of Section 2. 

The approach based on ACD estimations is depicted in Figure 4 as a regression line together with 95% confidence and prediction limits. The regression coefficient was *r* = 0.942 (*p* < 0.0001; slope of 0.676 with an intercept of −0.717) and the mean absolute error was 0.537. Such errors of logarithmic solubility around 0.5 are often obtained with drug solubility predictions of different methods. Limited prediction accuracy was already expected for the ACD in silico estimation of LSER parameters based on the previous comparison with IGC data. However, there can also be experimental factors contributing to limiting prediction accuracy by any theoretical prediction. It is, here, useful to consider the results of the residual solid form analysis that was determined as part of the solubility experiments. For example, the x-ray diffraction analysis of the residual solid revealed, for zafirlukast, at least one different solid form following equilibration, so we omitted the value with ethanol for the statistical evaluation due to a likely solvate formation. Moreover, the value of loratadine in heptane was omitted, and there were further cases in which likely the experimental complexity of the residual solid form was given. The residual solid XRPD revealed, for example, in the case of carvedilol, a changed solid form in dichloromethane after equilibration, which likely suggested a solvate formation.

Figure 5 shows the corresponding regression analysis of estimated values by the PSP approach, and the regression was slightly better with *r* = 0.958 (*p* < 0.0001; slope of 0.753 with an intercept of −0.634) and the mean absolute error yielded 0.481. Overall, the solubility predictions were reasonable, and it is well possible that higher precision would have been achieved by omission of all solubility data where solid form changes were detected during solubility equilibration. However, most solubility studies in the literature were not analyzing the residual solid form, so we decided to only omit clear outliers. As mentioned before, it is well possible that further precision can be achieved using more probe gases in IGC to determine PSPs, but the present approach (with a minimal dataset) was supposed to reflect a typical situation for practical solubility prediction, as it is feasible in the pharmaceutical industry.

The PSP framework can also be used for the estimation of the surface energy components of the studied drugs. However, there are practically no data on the total surface energies of drugs available in the literature. Instead, the dispersion components, *γ_d_*, were determined by the Dorris–Gray method [50]. This dispersion component is practically equivalent to our nonhydrogen-bonding component, *γ_VES_*. Thus, we may use Equation (29) in the equivalent form of Equation (36) and obtain the total surface energy of the drugs, *γ_tot_*:

(36) $$\gamma_{tot}=\gamma_{d}\frac{3.1V_{x}+E+S+2\sqrt{AB}}{3.1V_{x}+E+S}.$$

In a similar manner, the acidity and basicity components of the surface energy are obtained from Equation (27). The obtained surface energy components of the studied drugs are reported in Table 5.

Any reported value of surface energy contribution by IGC also involves a theoretical framework and, hence, the results in Table 5 are obtained surface estimates using IGC with the PSP approach. Due to the importance of surface energy contributions for pharmaceutics, one would expect that many poorly water-soluble drugs have been thoroughly characterized. However, values of surface energy contributions obtained by either contact angle measurements or IGC are only occasionally reported in the literature. Dispersive energy contribution was reported, for example, in the case of ketoconazole and values between about 40 and 50 mJ/m^2^ were obtained (based on IGC) depending on the surface disorder that was introduced by a milling process [51]. This result is in good agreement with our finding of *γ_d_* for ketoconazole. 

The surface energy estimations by the PSP approach can be further harnessed in future pharmaceutical studies on, for example, drug wettability. The PSP approach allows further theoretical options, like the estimation of cohesive energy density or equivalently, the solubility parameter and its components using Equations (1)–(4) and (13). These alternative characteristic descriptors of the drugs are reported in Table 6. As observed, the main component of the cohesive energy density in all studied drugs is the one reflecting dispersion forces, although the other components are by no means negligible. It is certainly an advantage of the PSP approach that a hydrogen-bonding contribution to cohesive energy density is differentiated according to acidity and basicity, which is missing in classical solubility parameter concepts.

## 5. Conclusions

PSPs were, for the first time, determined for drugs using inverse gas chromatography. Only a few probe gases were required to obtain estimates of PSP and even though more probe gases may have further increased the accuracy of the estimates, it was already possible to adequately apply the PSP of the model drugs to solubility data, as well as to predict components of surface energy. Less precise estimates were evidenced by an alternative LSER approach using pure in silico predictors. A clear advantage of the introduced PSP concept is that it allows for direct conversion to solubility parameters, as well as LSER parameters. Interesting is not only this conversion between different solvatochromic predictors by the PSP approach, but also the conversion of bulk to surface energies and vice versa. PSPs can therefore be seen as unified molecular descriptors, which advance classical solvatochromic parameters using a sound thermodynamic basis. Accordingly, there is much promise for diverse pharmaceutical applications from formulation science to biopharmaceutics.

## Figures and Tables

**Table 1:** Ultra performance liquid chromatographic (UPLC) analytics.

Compound	Gradient (A:B) (%)	Detection Wavelength (nm)
Carvedilol	90:10	241
Cyclosporine A	50:50	210
Ketoconazole	90:10	220
Loratadine	90:10	270
Simvastatin	30:70	230
Zafirlukast	40:60	225

**Table 2:** The linear solvation energy relationship (LSER)-type molecular descriptors of common solvents [7,20].

Solvent	*V_x_*	*E*	*S*	*A*	*B*
n-Hexane	0.954				
n-Heptane	1.095				
n-Octane	1.236				
n-Nonane	1.377				
n-Decane	1.518				
Cyclohexane	0.845	0.310	0.010		
Carbon tetrachloride	0.739	0.460	0.380		
Benzene	0.716	0.610	0.520		
Toluene	0.857	0.600	0.520		
Ethylbenzene	0.998	0.610	0.510		
Acetone	0.547	0.180	0.703		0.493
Methyl ethyl ketone	0.688	0.170	0.697		0.510
Ethyl acetate	0.747	0.110	0.618		0.450
n-Butyl acetate	1.028	0.071	0.597		0.449
Tetrahydrofuran	0.622	0.291	0.524		0.479
1,4-Dioxane	0.681	0.330	0.744		0.635
Chloroform	0.617	0.430	0.490	0.150	
Dichloromethane	0.494	0.390	0.574	0.100	
Methanol	0.308	0.280	0.439	0.430	0.470
Ethanol	0.449	0.250	0.419	0.370	0.480
1-Propanol	0.590	0.240	0.420	0.370	0.480
1-Butanol	0.731	0.220	0.420	0.370	0.480
1-Octanol	1.295	0.200	0.421	0.370	0.480
Isopropanol	0.590	0.210	0.366	0.330	0.560
2-Butanol	0.731	0.220	0.360	0.330	0.560
Cyclohexanol	0.904	0.461	0.557	0.320	0.570
Phenol	0.775	0.808	0.890	0.600	0.300
Ethylene glycol	0.508	0.400	0.900	0.580	0.780
Acetonitrile	0.404	0.241	0.900	0.040	0.330
Water	0.167	0.000	0.450	0.820	0.350

**Table 3:** The LSER descriptors for the six drugs as predicted by ACD software (ACD Percepta, Absolv. V.2016) compared with the partial solvation parameters (PSP) approach using experimental inverse gas chromatography (IGC) data.

Parameter	Carvedilol	Cyclosporine A	Ketoconazole	Loratadine	Simvastatin	Zafirlukast
LSER						
*Vx*	3.10	10.02	3.72	2.87	3.43	4.23
*E*	3.08	4.23	3.14	2.19	1.35	3.64
*S*_(ACD)_	3.00	10.16	3.76	2.09	2.29	4.09
*S*_(PSP)_	3.19	7.72	2.75	2.96	4.28	4.66
*A*_(ACD)_	0.62	1.25	0.00	0.00	0.31	0.85
*A*_(PSP)_	0.50	0.7 ± 0.1	0.00	0.00	0.20	0.71
*B*_(ACD)_	2.09	7.61	2.22	1.14	1.45	2.13
*B*_(PSP)_	1.45	4.5	0.7 ± 0.3	0.6 ± 0.2	0.7 ± 0.2	2.1

**Table 4:** Melting point temperatures and heats of fusion of the drugs.

Drug	*T^m^* (K)	Δ*H^m^* (kJ/mol)
Carvedilol	388.15 ± 0.12	47.36 ± 0.37
Cyclosporine A	n.a. *	n.a. *
Ketoconazole	422.29 ± 0.01	53.16 ± 0.59
Loratadine	407.29 ± 0.05	27.97 ± 0.18
Simvastatin	412.21 ± 0.06	29.16 ± 0.83
Zafirlukast	467.95 ± 0.1	19.6 ± 1.7

* No data were available due to a primarily amorphous solid.

**Table 5:** The surface energy components of the drugs obtained by the PSP/LSER method.

Drug	*γ_d_* (mJ/m^2^)	*γ_hb_* (mJ/m^2^)	*γ_tot_* (mJ/m^2^) *	*γ_a_*	*γ_b_*
Carvedilol	47.83	5.13	52.96	1.51	4.37
Cyclosporine A	13.19	1.09	14.28	0.21	1.38
Ketoconazole	45.86	0.00	45.86	0.00	1.84
Loratadine	41.6	0.00	41.60	0.00	1.78
Simvastatin	57.36	2.64	60.00	0.71	2.47
Zafirlukast	49.43	5.64	55.07	1.64	4.85

* Total surface energy (*γ_tot_*).

**Table 6:** The cohesive energy (solubility–parameter) components of the drugs obtained by the PSP/LSER method.

Drug	*σ_d_* (MPa^0.5^)	*σ_p_* (MPa^0.5^)	*σ_tot_* * (MPa^0.5^)	*σ_hb_* (MPa^0.5^)	*σ_Ga_* (MPa^0.5^)	*σ_Gb_* (MPa^0.5^)
Carvedilol	19.75	9.91	23.55	8.14	3.92	6.68
Cyclosporine A	18.04	8.44	21.12	7.03	2.54	6.44
Ketoconazole	19.52	8.45	21.27	0.00	0.00	4.26
Loratadine	19.11	9.87	21.51	0.00	0.00	4.45
Simvastatin	17.82	10.65	20.96	2.89	2.30	4.31
Zafirlukast	19.60	10.33	23.92	9.01	4.03	6.94

* Square root of total cohesive energy density (equivalent to *δ_tot_*).
